# Three-dimensional bioprinted organoids: advances and clinical translation

**DOI:** 10.1093/rb/rbag142

**Published:** 2026-06-30

**Authors:** Yi Zhang, Zhen Huang, Beichen Wang, Bing Li, Yunfeng Yang

**Affiliations:** Department of Orthopedics, Tongji Hospital, School of Medicine, Tongji University, Shanghai 200092, China; Department of Orthopedics, The First Affiliated Hospital, Fujian Medical University, Fuzhou 350005, China; Department of Orthopedics, National Regional Medical Center, Binhai Campus of the First Affiliated Hospital, Fujian Medical University, Fuzhou 350212, China; Department of Orthopedics, Ruijin Hospital, Shanghai Jiao Tong University School of Medicine, Shanghai 200025, China; Department of Orthopedics, Ruijin Hospital, Shanghai Jiao Tong University School of Medicine, Shanghai 200025, China; Department of Orthopedics, Ruijin Hospital, Shanghai Jiao Tong University School of Medicine, Shanghai 200025, China

**Keywords:** 3D bioprinting, organoids, clinical translation, bio-inks, personalized medicine, tissue engineering, regenerative medicine

## Abstract

Three-dimensional (3D) bioprinting enables the precise fabrication of complex tissue constructs by depositing living cells, biomaterials, and bioactive factors in a spatially controlled manner. Concurrently, organoids—self-organizing 3D cellular structures that recapitulate key aspects of organ architecture and function—have revolutionized biomedical research by providing physiologically relevant models for disease modeling, drug screening, and regenerative medicine. The convergence of 3D bioprinting and organoid technology represents a significant advancement, offering new opportunities for personalized medicine and tissue engineering. This review examines the current state of 3D bioprinted organoids, encompassing bioprinting techniques, bio-ink development, and diverse clinical applications spanning oncology (patient-derived tumor organoids for predicting therapy response in selected gastrointestinal cancers), regenerative medicine (bioprinted bone, cartilage, cardiac, and skin constructs), drug discovery (hepatotoxicity and nephrotoxicity screening platforms), and personalized therapeutics. We discuss technological advances supporting translational development, while distinguishing clinically validated patient-derived tumor organoid evidence from preclinical and proof-of-concept regenerative applications, and critically analyze the challenges that must be addressed for widespread clinical adoption. Finally, we provide perspectives on future directions, including the integration of artificial intelligence, multiorganoid systems, and regulatory pathways that will shape the next generation of 3D bioprinted organoids for clinical use.

## Graphical abstract

**Graphical Abstract:**
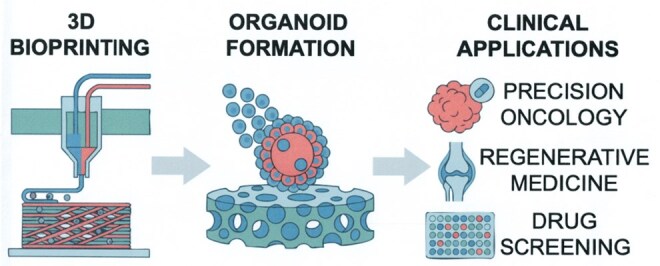

## Introduction

The field of regenerative medicine and tissue engineering has witnessed remarkable advances over the past decade, driven by the intersection of multiple technological innovations [1, 2]. Among these, three-dimensional (3D) bioprinting and organoid technology stand out as two of the most promising approaches for creating functional tissue constructs that can bridge the gap between laboratory research and clinical applications [3, 4].

Organoids are self-organizing 3D cellular structures derived from stem cells or organ-specific progenitor cells that recapitulate key architectural and functional features of their corresponding organs [5]. Since the pioneering work demonstrating intestinal organoid formation from single Lgr5+ stem cells [6], organoid technology has rapidly expanded to encompass virtually every organ system, including brain [7], liver [8, 9], kidney [10], lung, heart, and pancreas. These miniature organ-like structures have proven invaluable for studying human development, modeling diseases, and screening therapeutic compounds in a physiologically relevant context.

However, conventional organoid culture systems face significant limitations that hinder their clinical translation. Traditional organoid formation relies on spontaneous self-organization within extracellular matrix (ECM) gels, resulting in variable sizes, shapes, and cellular compositions [11]. This inherent heterogeneity poses challenges for standardization, reproducibility, and scalability—all critical requirements for clinical applications. Furthermore, organoids cultured using conventional methods often lack proper vascularization, limiting nutrient and oxygen diffusion and constraining their growth to submillimeter dimensions [12].

3D bioprinting technology offers elegant solutions for many of these limitations [1, 13]. By precisely depositing cells, biomaterials, and bioactive factors in predetermined spatial patterns, bioprinting enables the fabrication of tissue constructs with controlled architecture, reproducible dimensions, and incorporated vascular networks [14, 15]. The marriage of organoid biology with bioprinting engineering thus represents a powerful synergy that can overcome the individual limitations of each approach.

The clinical translation of 3D bioprinted organoids encompasses multiple application domains. In oncology, patient-derived tumor organoids can be bioprinted to create personalized cancer models for drug sensitivity testing, enabling precision medicine approaches that match patients with optimal therapeutic regimens [16, 17]. In regenerative medicine, bioprinted organoids incorporating autologous cells offer the potential for transplantable tissue constructs that can repair or replace damaged organs [18]. In drug discovery, standardized bioprinted organoid platforms provide more predictive models of human drug responses compared to traditional two-dimensional cell cultures or animal models [19].

This review aims to provide a comprehensive overview of the current state of 3D bioprinted organoids, with a particular focus on clinical applications. We begin by reviewing the fundamental principles and techniques of 3D bioprinting relevant to organoid fabrication. We then examine the critical role of bio-inks—the printable materials that encapsulate cells and provide structural support—in determining organoid viability and function. Subsequently, we explore the diverse clinical applications of bioprinted organoids across different medical specialties. We discuss the challenges and barriers to clinical translation, and conclude with perspectives on future developments that will shape this rapidly evolving field.

While several excellent reviews have addressed bioprinting technologies or organoid biology independently, this review uniquely focuses on their integration from a translational-readiness perspective. Specifically, we synthesize how technique selection, bio-ink design, organoid biology, application evidence, and regulatory/manufacturing barriers jointly determine whether reported systems are clinically actionable or remain preclinical.

## Fundamentals of 3D bioprinting technology

### Overview of bioprinting techniques

**Figure 1:**
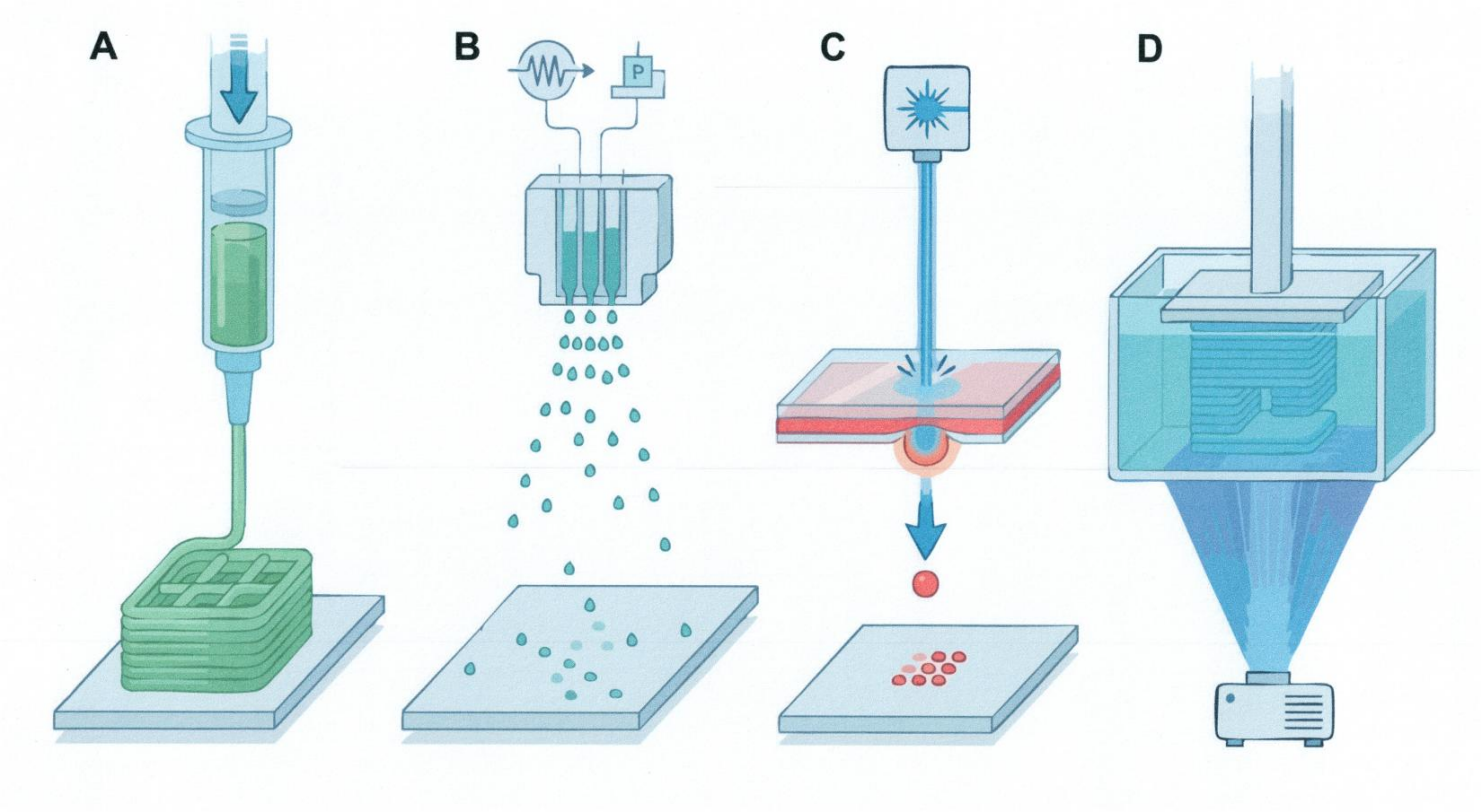
Overview of 3D bioprinting modalities for organoid fabrication, mapped to ISO/ASTM 52900:2021 process terminology. (**A**) Extrusion-based bioprinting using pneumatic or mechanical pressure to deposit continuous bio-ink filaments. (**B**) Jetting-based bioprinting (inkjet variant) employing thermal or piezoelectric actuation to eject discrete bio-ink droplets onto a substrate. (**C**) Jetting-based bioprinting (laser-assisted variant) utilizing laser pulses to propel bio-ink droplets from a donor ribbon without nozzle contact. (**D**) Vat photopolymerization-based bioprinting (DLP variant) using projected light patterns to simultaneously photocrosslink entire layers of cell-laden resin.

3D bioprinting encompasses a family of additive manufacturing techniques adapted for the precise deposition of living cells and biomaterials [1, 13]. Using ISO/ASTM 52900:2021 additive manufacturing terminology, the modalities discussed here mainly map to material extrusion, material jetting or jetting-based droplet printing, and vat photopolymerization (Figure 1; Table 1). Each category offers distinct advantages and limitations in terms of resolution, cell viability, printing speed, and material compatibility [2, 20].

**Table 1:** Comparison of 3D bioprinting techniques for organoid fabrication.

Technique	Resolution	Cell viability (%)	Printing speed	Bio-ink viscosity	Key applications
Extrusion-based	100–500 μm	40–90	Moderate	High (30–10^7^ mPa·s)	Bone, cartilage, skin constructs
Jetting-based (inkjet)	20–100 μm	80–95	Fast	Low (3–30 mPa·s)	Gradient structures, drug screening
Jetting-based (LAB)	10–50 μm	>95	Slow	Low–Medium	High-precision patterning
Vat photopolymerization	<50 μm	70–95	Variant-dependent	Low–Medium	Vascular networks, complex geometries

Values are representative and formulation-, nozzle-, shear-rate-, light-dose-, and instrument-dependent.

#### Extrusion-based bioprinting

Extrusion-based bioprinting is the most widely adopted technique for fabricating tissue constructs due to its versatility, scalability, and compatibility with a broad range of bio-inks [21, 22]. In this approach, bio-ink is dispensed through a nozzle or needle under pneumatic, piston-driven, or screw-driven pressure, creating continuous filaments that are deposited layer-by-layer to build 3D structures.

The key advantages of extrusion bioprinting include its ability to handle high cell densities (typically 10^6^–10^8^ cells/mL), compatibility with viscous hydrogels, and relatively low equipment costs. Modern extrusion bioprinters can achieve printing resolutions of 100–500 μm, which are sufficient for many tissue engineering applications [18]. However, the shear stresses experienced by cells during extrusion can compromise cell viability, particularly when using narrow nozzles or highly viscous bio-inks [22].

Beyond mechanical shear stresses during extrusion, the subsequent crosslinking mechanism significantly influences cell viability in extrusion-based bioprinting. Chemical crosslinking agents such as glutaraldehyde can stabilize hydrogel structures but are generally unsuitable for cell-laden constructs except in carefully controlled or acellular postprocessing contexts because of dose-dependent cytotoxicity. Ionic crosslinking, commonly employed for alginate-based bio-inks using calcium chloride solutions, is generally well tolerated by cells but may provide limited long-term mechanical stability. Photocrosslinking enables rapid gelation with excellent spatial control, though optimization of photoinitiator concentration and light exposure duration is essential to minimize radical-mediated cellular damage. Enzymatic crosslinking, such as transglutaminase-mediated gelation of gelatin-based bio-inks, offers particularly gentle conditions favorable for maintaining high cell viability, albeit with slower gelation kinetics. Thermal crosslinking strategies exploit temperature-dependent sol–gel transitions, as seen in gelatin and Pluronic F-127 systems, providing mild processing conditions but often resulting in mechanically weak constructs that require secondary crosslinking [22, 23].

#### Jetting-based bioprinting

Jetting-based bioprinting encompasses techniques that deposit cell-laden bio-ink as discrete droplets onto a substrate, including inkjet bioprinting and laser-assisted bioprinting [1, 24]. This category is unified by the fundamental principle of generating and precisely placing individual bio-ink droplets to build 3D structures.

Inkjet bioprinting employs thermal or piezoelectric actuators to eject droplets of bio-ink with high spatial precision [1]. Thermal inkjet systems generate vapor bubbles through localized heating that propel droplets from the nozzle orifice, while piezoelectric systems use mechanical deformation of a piezoelectric element to create pressure pulses. This technique offers higher resolution (20–100 μm) and faster printing speeds compared to extrusion methods, enabling precise control over the spatial distribution of multiple cell types and growth factors. However, inkjet bioprinting is limited to low-viscosity bio-inks (often approximately 3–30 mPa·s, depending on the printhead), which may not provide adequate structural support for organoid formation [23].

Several key parameters critically affect cell viability in jetting-based bioprinting. Polymer concentration directly influences droplet formation dynamics and bio-ink viscosity, with higher concentrations increasing viscous dissipation during droplet ejection [25]. Droplet velocity, typically ranging from 1 to 10 m/s, subjects cells to impact forces upon substrate contact that can compromise membrane integrity at excessive speeds [26]. Droplet volume, often in the subnanoliter to picoliter range (1–300 pL), determines the number of cells per droplet and the resolution of spatial patterning. Additionally, nozzle diameter, pulse frequency, and substrate surface properties all contribute to the fidelity and biological outcomes of jetting-based bioprinting [24].

Laser-assisted bioprinting (LAB) represents a nozzle-free variant of jetting-based bioprinting that utilizes focused laser pulses to transfer bio-ink from a donor ribbon onto a receiving substrate [2]. The laser energy vaporizes a sacrificial absorption layer, generating a cavitation bubble that propels a droplet of bio-ink toward the substrate without direct contact between the bio-ink and the laser. This noncontact approach minimizes mechanical stress on cells and enables high-resolution printing (10–50 μm) with excellent cell viability (>95%). Key parameters affecting LAB outcomes include laser energy density, bio-ink film thickness, and the gap distance between donor ribbon and receiving substrate. Despite these advantages, LAB systems are considerably more expensive than inkjet printers and have lower throughput due to the point-by-point scanning required for pattern generation, limiting widespread adoption for large-scale organoid fabrication [27].

#### Vat photopolymerization-based bioprinting

Vat photopolymerization-based bioprinting employs light to selectively photocrosslink liquid photopolymer resins containing cells within a vat or reservoir [15, 28]. This category encompasses several variants, including stereolithography with point-by-point laser scanning, digital light processing (DLP) with layer-at-once projection via a digital micromirror device, continuous liquid interface production for accelerated printing, and emerging volumetric bioprinting approaches that use tomographic light projection to fabricate entire structures within seconds.

The photopolymerization mechanisms underlying vat-based bioprinting primarily include free-radical chain polymerization of (meth)acrylate monomers and thiol-ene step-growth click chemistry [28]. Free-radical polymerization is the most widely used mechanism, offering rapid gelation and high mechanical strength, but is susceptible to oxygen inhibition and can generate network heterogeneity with associated shrinkage stress. Thiol-ene click chemistry provides an attractive alternative with reduced oxygen inhibition, lower shrinkage stress, and more homogeneous network formation, though it requires careful stoichiometric balancing of thiol and ene functional groups.

The choice of photoinitiator is critical for both printing fidelity and cell viability. UV-activated photoinitiators such as Irgacure 2959 have been widely used but raise concerns about UV-induced DNA damage in encapsulated cells. Visible-light photoinitiators, including lithium phenyl-2,4,6-trimethylbenzoylphosphinate and eosin Y/triethanolamine systems, enable bioprinting at longer wavelengths (405–530 nm) with reduced phototoxicity [29]. Photo-absorbers such as tartrazine and Ponceau 4R play an essential role in controlling light penetration depth and *z*-axis resolution, preventing overcuring of previously deposited layers. Photocurable bio-ink formulations include GelMA, polyethylene glycol diacrylate, silk fibroin-methacrylate, hyaluronic acid-methacrylate, and their hybrid blends, each offering distinct mechanical properties and biological performance [28].

DLP-based bioprinting, in particular, offers exceptionally high resolution (<50 μm) and rapid fabrication speeds because entire layers are cured simultaneously. Parameters that critically affect cell viability include light intensity, exposure time per layer, photoinitiator concentration and type, absorption wavelength, and the extent of radical-mediated cytotoxicity during polymerization [15].

### Key parameters influencing bioprinting quality

The successful fabrication of bioprinted organoids depends on careful optimization of multiple printing parameters [30]. These include bio-ink properties (viscosity, crosslinking kinetics, cell density), printing parameters (nozzle diameter, printing speed, extrusion pressure), and environmental conditions (temperature, humidity, sterility).

The interplay between bio-ink viscosity and shear stress is particularly critical. Higher viscosity bio-inks provide better shape fidelity after printing but subject cells to greater shear stress during extrusion, potentially compromising viability [22]. Shear-thinning bio-inks that exhibit reduced viscosity under shear stress offer a favorable balance, enabling smooth extrusion while maintaining structural integrity after deposition [31].

## Bio-inks for organoid bioprinting

### Essential properties of bio-inks

Bio-inks serve as the carrier medium for cells during bioprinting and provide the structural and biochemical environment necessary for organoid development [23, 32]. An ideal bio-ink for organoid bioprinting must satisfy multiple, often competing, requirements. First, the bio-ink must possess appropriate rheological properties to enable consistent extrusion or deposition while maintaining shape fidelity after printing, typically requiring shear-thinning behavior and rapid postprinting gelation or crosslinking [22, 31]. Equally important, the bio-ink components and crosslinking chemistry must be nontoxic to encapsulated cells and support cell survival, proliferation, and differentiation over extended culture periods [23]. Beyond basic biocompatibility, an effective bio-ink should recapitulate key features of the native ECM, including appropriate mechanical stiffness, cell-adhesive ligands, and matrix remodeling capabilities [33]. Finally, for many applications, the bio-ink scaffold should degrade at a rate that matches tissue formation, allowing the organoid’s own ECM to progressively replace the synthetic scaffold [21].

### Natural polymer-based bio-inks

**Figure 2:**
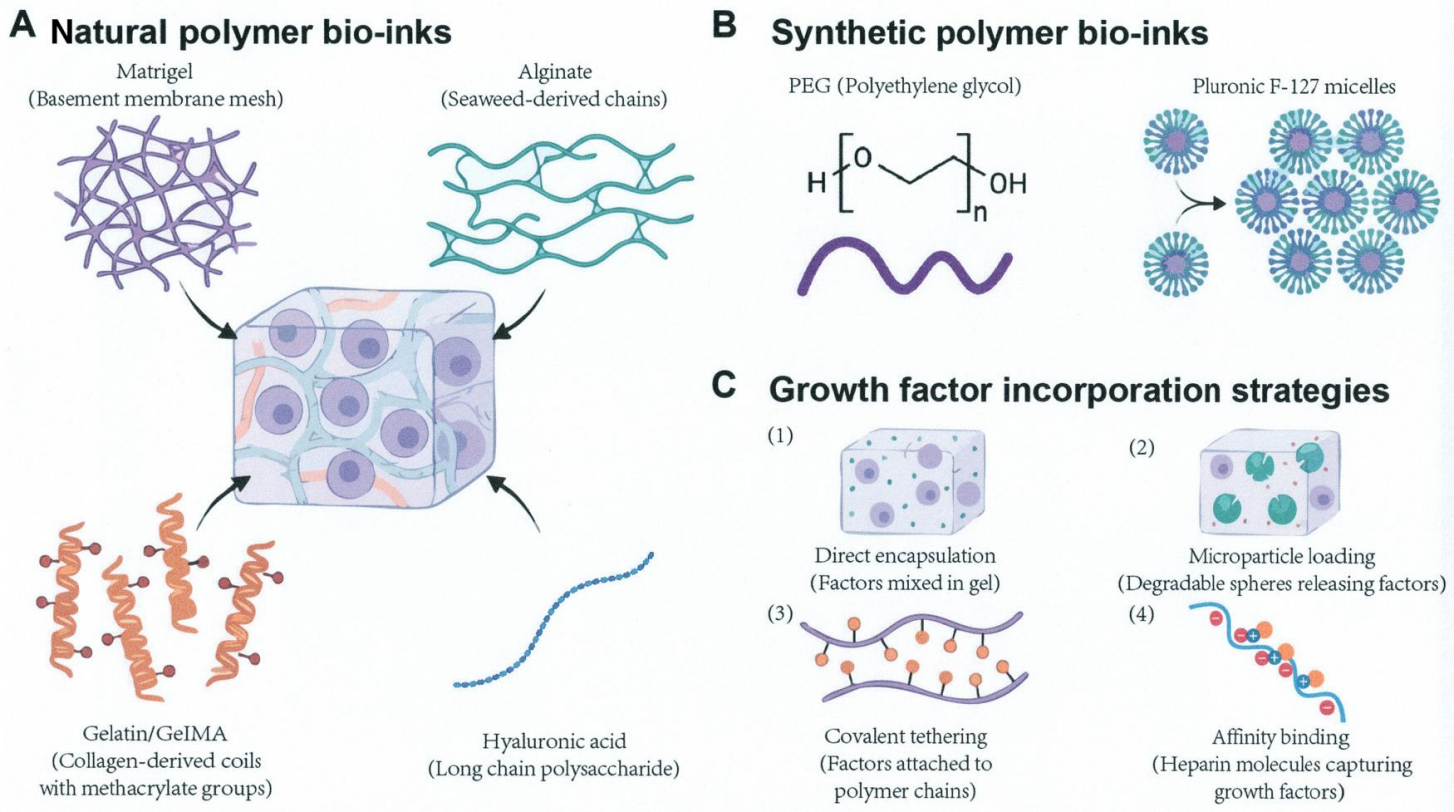
Bio-ink components and design strategies for organoid bioprinting. (**A**) Natural polymer-based bio-inks including Matrigel, alginate, gelatin, and hyaluronic acid. (**B**) Synthetic polymer-based bio-inks with tunable properties. (**C**) Growth factor incorporation strategies: direct encapsulation, microparticle loading, covalent tethering, and affinity binding.

Natural polymers derived from biological sources offer inherent biocompatibility and often contain cell-adhesive motifs that promote cell attachment and function (Figure 2) [32].

#### Matrigel and basement membrane extracts

Matrigel, a basement membrane extract derived from Engelbreth-Holm-Swarm mouse sarcoma, has been the gold standard for organoid culture due to its rich composition of laminin, Collagen IV, entactin, and growth factors [5, 6]. However, Matrigel poses significant challenges for bioprinting and clinical translation. Its batch-to-batch variability compromises reproducibility, its murine origin raises concerns about xenogeneic contamination, and its temperature-sensitive gelation (liquid at 4°C, gelling at 37°C) complicates printing workflows.

Several strategies have been developed to adapt Matrigel for bioprinting applications, including blending with alginate or other printable polymers, optimizing temperature-controlled printing systems, and developing bioprinted scaffolds that can be subsequently infused with Matrigel [11].

#### Alginate-based bio-inks

Alginate, a polysaccharide derived from brown seaweed, offers excellent printability due to its rapid ionic crosslinking with divalent cations (Ca^2+^, Ba^2+^) [32]. The mechanical properties of alginate hydrogels can be tuned by varying polymer concentration, molecular weight, and crosslinker concentration.

However, alginate lacks cell-adhesive motifs, necessitating modification with arginine–glycine–aspartate (RGD) peptides or blending with other ECM components. Alginate-based bio-inks have been used in selected bioprinting contexts, but organoid-specific applications require matching the alginate formulation to cell-adhesion and maturation needs [32, 34].

#### Gelatin and gelatin methacrylate

Gelatin, derived from collagen hydrolysis, retains cell-binding sequences (RGD) and matrix metalloproteinase (MMP)-sensitive degradation sites that promote cell adhesion and matrix remodeling [35]. Gelatin methacrylate (GelMA), created by functionalizing gelatin with methacrylate groups, enables photocrosslinking under UV or visible-light exposure.

GelMA-based bio-inks have become increasingly popular for organoid bioprinting due to their favorable combination of bioactivity and tunable mechanical properties [29]. The degree of methacrylation and polymer concentration can be adjusted to optimize both printability and organoid formation.

#### Hyaluronic acid-based bio-inks

Hyaluronic acid (HA), a major component of the native ECM, plays important roles in cell signaling, migration, and tissue hydration [31]. HA-based bio-inks offer excellent biocompatibility and can be modified with various crosslinkable groups (methacrylate, thiol, adamantane) to enable different gelation mechanisms.

HA bio-inks are particularly relevant for organoids derived from tissues with high HA content, such as brain, cartilage, and certain tumors. The viscoelastic properties of HA hydrogels can influence organoid morphogenesis and differentiation [33].

### Synthetic polymer-based bio-inks

Synthetic polymers offer advantages in terms of batch consistency, tunable properties, and defined composition, although they typically lack the inherent bioactivity of natural polymers [32]. A comprehensive comparison of bio-ink materials and their properties is provided in Table 2.

**Table 2:** Summary of bio-ink materials and their properties for organoid bioprinting.

Bio-ink material	Origin	Crosslinking	Key properties	Organoid applications
Matrigel	Natural (EHS tumor)	Temperature-induced gelation	ECM-rich, high bioactivity	Intestinal, liver, tumor organoids
Alginate	Natural (seaweed)	Ionic (Ca^2+^)	Rapid gelation, low cost	Cartilage, bone, encapsulation
GelMA	Semi-synthetic	Photo (UV/visible)	Tunable stiffness, RGD motifs	Cartilage, cardiac, vascular
Hyaluronic acid	Natural (ECM)	Various	Viscoelastic, CD44 binding	Neural, cartilage organoids
PEG-based	Synthetic	Photo/chemical	Defined, customizable	Controlled microenvironments
dECM	Natural (tissue)	Thermal/pH; optional chemical/photo	Tissue-specific cues	Tissue-matched organoids

#### Polyethylene glycol

Polyethylene glycol (PEG)-based hydrogels provide a “blank slate” that can be systematically functionalized with bioactive motifs [21]. The bioinert PEG backbone resists protein adsorption and cell attachment, enabling precise control over cell-material interactions through incorporated peptides or proteins.

PEG-based bio-inks have been used for bioprinting organoids with defined microenvironments, where specific combinations of adhesive ligands and degradable crosslinks guide organoid formation and maturation [23].

#### Pluronic F-127

Pluronic F-127, a triblock copolymer of polyethylene oxide and polypropylene oxide, exhibits thermoreversible gelation—forming a gel above its critical micelle temperature (approximately 20°C) and liquefying upon cooling [14]. This property makes Pluronic F-127 useful as a sacrificial material for creating perfusable channels within bioprinted constructs.

### Growth factor incorporation strategies

The incorporation of growth factors and other bioactive molecules into bio-inks is essential for directing organoid development and maturation [5, 12]. Several strategies have been developed for growth factor delivery within bioprinted constructs. The simplest approach, direct encapsulation, involves mixing growth factors directly into the bio-ink before printing; while this provides immediate bioavailability, it may result in rapid diffusion out of the construct. To achieve more sustained release, growth factors can be encapsulated within degradable microparticles that gradually release their cargo as the particles erode [33]. Alternatively, covalent tethering—chemically conjugating growth factors to the bio-ink polymer—enables localized presentation while preventing diffusion. A fourth strategy exploits affinity-based sequestration, in which bio-inks are functionalized with heparin or other glycosaminoglycans that bind and retain heparin-binding growth factors within the construct.

Key growth factors for organoid bioprinting include Wnt agonists (R-spondin, CHIR99021), epidermal growth factor family members, fibroblast growth factors (FGFs), bone morphogenetic proteins (BMPs), and Noggin, with specific combinations tailored to the target organoid type [5, 6].

## Clinical applications of 3D bioprinted organoids

### Personalized cancer medicine

#### Patient-derived tumor organoids for drug screening

**Figure 3:**
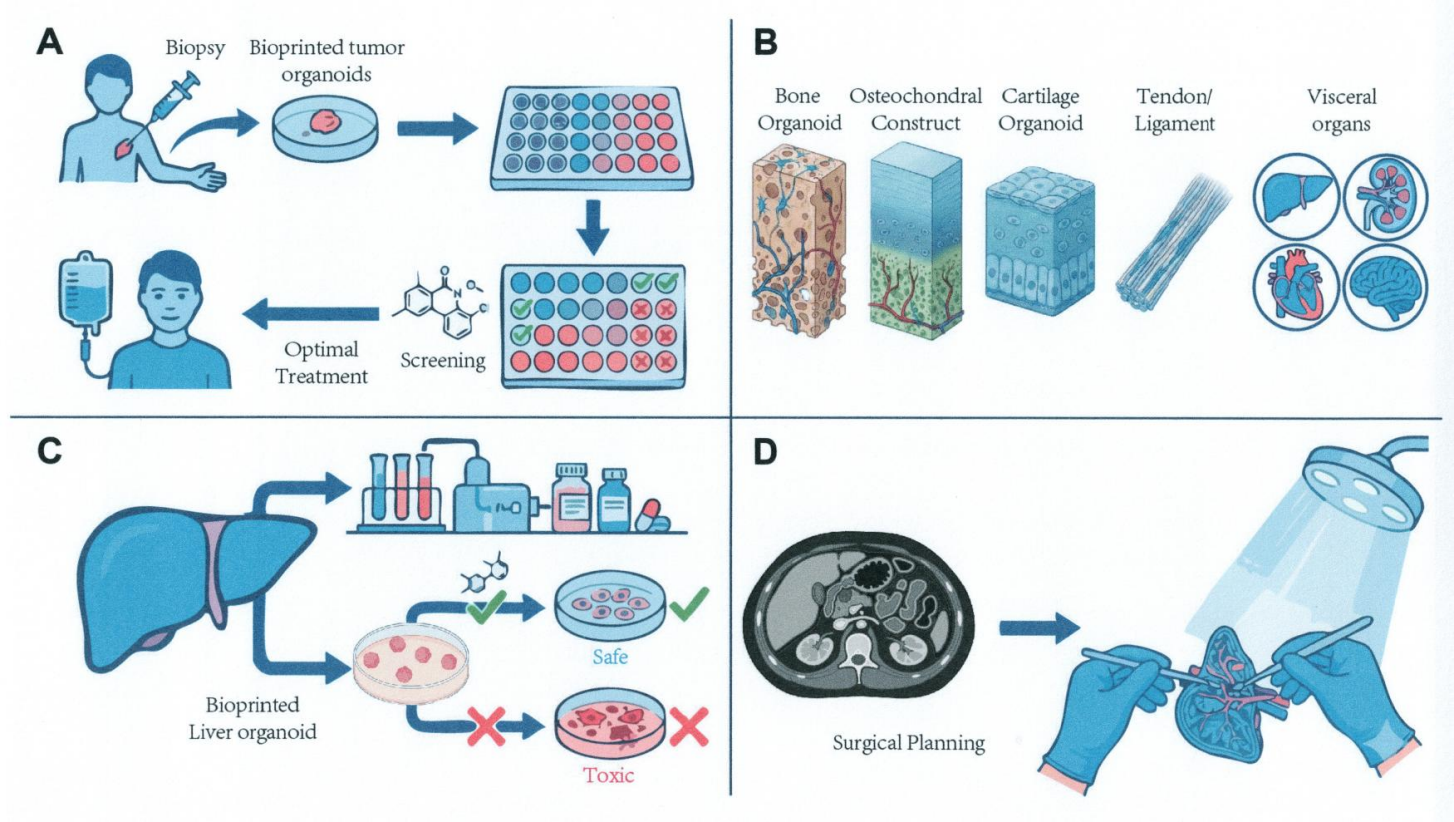
Current and emerging translational applications of 3D bioprinted organoids. (**A**) Personalized cancer medicine: patient-derived tumor organoids for drug sensitivity testing and precision oncology. (**B**) Preclinical regenerative medicine applications with emphasis on musculoskeletal system (bone, osteochondral, cartilage, tendon/ligament organoids) and visceral organs (liver, kidney, cardiac, neural). (**C**) Drug discovery and toxicology screening platforms. (**D**) Surgical planning models and tissue engineering for orthopedics and plastic surgery.

Patient-derived tumor organoids (PDTOs) have emerged as powerful tools for predicting individual patient responses to anticancer therapies (Figure 3A) [16, 36]. By establishing organoids from tumor biopsies, clinicians can test multiple drug candidates or combinations *ex vivo* in research or selected precision-oncology settings before initiating treatment, potentially improving response rates and avoiding ineffective therapies.

3D bioprinting enhances PDTO applications in several important ways. First, bioprinting enables standardized organoid production by generating uniform organoid arrays with consistent size and cell number, substantially improving the reproducibility of drug response assays [11]. Second, automated bioprinting platforms support high-throughput screening by producing hundreds to thousands of organoids per hour, facilitating comprehensive drug library screening [19]. Third, bioprinting facilitates tumor microenvironment modeling by enabling coculture of tumor organoids with stromal cells, immune cells, and vascular networks, creating more physiologically relevant models that better capture the complexity of *in vivo* tumor biology [37].

Several landmark clinical studies have validated the predictive power of PDTOs. Ooft *et al.* conducted a prospective study demonstrating that organoid drug sensitivity testing predicted clinical response to irinotecan-based therapy in more than 80% of metastatic colorectal cancer patients [17]. For locally advanced rectal cancer, Yao *et al.* established that organoid-based testing could predict chemoradiation responses with 84% accuracy, 78% sensitivity, and 92% specificity [38]. Ganesh *et al.* created a living biobank of rectal cancer organoids that demonstrated strong correlations between organoid and patient responses to chemoradiation [39]. In colorectal peritoneal metastases, Narasimhan *et al.* demonstrated that organoid drug screening could identify *ex vivo* drug sensitivities and inform therapy changes in a subset within clinically actionable timeframes [40]. These studies validate PDTO-guided testing rather than the independent clinical added value of bioprinting, which still requires head-to-head validation against conventional organoid formats.

#### Bioprinted tumor models for immunotherapy development

The advent of cancer immunotherapy has created demand for preclinical models that accurately recapitulate tumor-immune interactions [37]. Bioprinted tumor organoid models incorporating patient-matched immune cells enable evaluation of checkpoint inhibitors, chimeric antigen receptor-T cells, and other immunotherapies in a personalized context.

Bioprinting facilitates the spatial organization of tumor organoids within a stromal matrix containing immune cell populations, mimicking the architecture of the tumor microenvironment. Dijkstra *et al.* pioneered the coculture of tumor organoids with autologous peripheral blood lymphocytes, demonstrating successful generation of tumor-reactive T cells from both colorectal and non-small cell lung cancer patients [37]. These models have shown promise for predicting immunotherapy responses and identifying resistance mechanisms [19].

### Regenerative medicine and tissue engineering

#### Bone and osteochondral tissues

Bone defects resulting from trauma, tumor resection, or degenerative diseases represent a significant clinical burden in orthopedic surgery [41]. Traditional treatments including autografts and allografts face limitations in availability, donor site morbidity, and immunogenicity. 3D bioprinting offers a promising preclinical alternative by enabling the fabrication of patient-specific bone constructs with controlled architecture, porosity, and mechanical properties [18, 42, 43].

Recent advances in bone organoid engineering have yielded promising preclinical results. Su and colleagues developed a novel approach using bone matrix-inspired hydroxyapatite hybrid bio-inks to engineer large-scale self-mineralizing bone organoids, demonstrating enhanced osteogenic differentiation and mineralization [44]. Their comprehensive review on bone/cartilage organoid engineering provides valuable insights into construction strategies and potential translational applications [43]. Additionally, the concept of small joint organoids fabricated through 3D bioprinting has emerged as a notable platform for studying joint biology and developing therapeutic interventions for joint diseases [45].

The integrated tissue-organ printing (ITOP) system has demonstrated the capacity to produce human-scale bone constructs with structural integrity, incorporating microchannels that facilitate nutrient diffusion and cell survival [18]. Light-based bioprinting of DNA hydrogels represents an innovative approach for bone regeneration with implications for bone organoid development [46]. These constructs have shown successful integration and new bone formation in preclinical animal models. Key considerations for bone bioprinting include the selection of appropriate bio-ink compositions, cell sources, and growth factor delivery strategies. Bio-ink formulations typically incorporate calcium phosphate ceramics such as hydroxyapatite and β-tricalcium phosphate combined with biodegradable polymers to provide both osteoconductivity and printability [47]. Cell sources commonly include mesenchymal stem cells (MSCs), osteoblasts, and endothelial cells for vascularization [41]. Growth factor delivery, particularly BMP-2 and vascular endothelial growth factor (VEGF), can be incorporated through direct encapsulation or controlled-release systems to enhance bone regeneration [42].

Osteochondral defects affecting both articular cartilage and underlying subchondral bone present particular challenges due to the need to regenerate two distinct tissue types with different compositions and mechanical properties [48]. Bioprinting enables the fabrication of gradient structures that transition from cartilage-like properties at the surface to bone-like properties at the base, mimicking the native osteochondral interface.

A particularly innovative approach is the engineering of osteo-callus organoids developed by Ouyang and colleagues, which recapitulates the endochondral ossification process [49]. Using DLP printing technology combined with stepwise induction of MSC-loaded hydrogel microspheres, they achieved rapid bone regeneration within just 4 weeks in large bone defects in rabbits—significantly faster than traditional tissue engineering approaches requiring 2–3 months. This developmental engineering strategy, which mimics the natural callus formation process, represents an important advance in bone regeneration approaches. Furthermore, their airflow-assisted 3D bioprinting method enables the fabrication of heterogeneous microspheroidal organoids with excellent resolution and spatial organization [50].

Recent studies have demonstrated successful regeneration of osteochondral defects using bioprinted biphasic constructs incorporating chondrocytes in the superficial layer and osteogenic cells in the deep layer [41].

#### Articular cartilage

Articular cartilage damage from osteoarthritis, trauma, or sports injuries affects millions of patients worldwide and represents a major focus of orthopedic tissue engineering [51]. The avascular nature of cartilage limits its intrinsic healing capacity, making bioprinted cartilage constructs an attractive therapeutic option.

3D bioprinting of hyaline articular cartilage requires careful consideration of the zonal organization present in native tissue, including superficial, transitional, and deep zones with distinct cellular phenotypes and ECM compositions [51]. Bioprinting strategies for cartilage regeneration encompass several complementary approaches. Zonal bioprinting aims to create layered constructs that recapitulate the superficial, transitional, and deep zones of articular cartilage with zone-specific cell densities and matrix compositions. Chondrocyte-laden bio-inks encapsulate primary chondrocytes or MSC-derived chondroprogenitors in hydrogels such as GelMA, alginate, or hyaluronic acid, providing both structural support and biological cues for chondrogenic differentiation [29, 32]. Postprinting mechanical conditioning through dynamic compression or hydrostatic pressure further promotes chondrogenic differentiation and ECM production, enhancing the functional properties of the resulting constructs.

A breakthrough in cartilage organoid research comes from Bai and colleagues, who developed a robust cartilaginous organoid system from human expanded pluripotent stem cells [52]. This system enables systematic drug screening and mechanistic studies. Notably, through screening 2040 Food and Drug Administration (FDA)-approved drugs using their organoid platform, they identified alpha2-adrenergic receptor signaling as a therapeutic target for cartilage regeneration [53]. The alpha-adrenergic receptor antagonist phentolamine was found to stimulate chondrogenesis while repressing hypertrophy, and demonstrated protective effects against osteoarthritis in preclinical models. This represents an excellent example of how organoid technology can accelerate drug discovery for cartilage diseases.

Clinical translation of bioprinted cartilage faces challenges including achieving adequate mechanical strength for load-bearing applications and ensuring long-term integration with native tissue [48].

#### Tendon and ligament

Tendon and ligament injuries, common in both athletic and aging populations, often result in incomplete healing with scar tissue formation that compromises mechanical function [54]. The highly organized, anisotropic structure of these tissues—characterized by aligned collagen fibers—requires specialized bioprinting approaches.

3D prestress bioprinting has emerged as an effective strategy for creating tissues with aligned fiber architecture [55]. This technique applies sustained tensile stress during printing and crosslinking, resulting in molecular chain orientation that mimics native tendon structure. Bioprinted tendon constructs using this approach have demonstrated improved mechanical properties and cellular alignment compared to conventional bioprinting methods.

Decellularized tendon extracellular matrix (dECM) has been explored as a bio-ink component to provide tissue-specific biochemical cues [54]. These dECM-based bio-inks retain native growth factors and structural proteins that promote tenocyte differentiation and tenogenic gene expression.

In ligament regeneration, Ouyang and colleagues demonstrated the long-term effects of knitted silk-collagen sponge scaffolds on anterior cruciate ligament (ACL) reconstruction [56]. Their innovative scaffold design features an “internal-space-preservation” property that enables satisfactory regeneration of ligament tissue, while also effectively protecting joint cartilage from osteoarthritis progression—addressing a common complication following ACL reconstruction.

Challenges in tendon bioprinting include achieving the high mechanical strength required for functional load transfer and promoting integration at the tendon–bone interface (enthesis).

#### Visceral organ organoids

Bioprinted organoids of visceral organs have made technical progress toward translational modeling, but organ-replacement applications remain preclinical despite the critical shortage of donor organs for transplantation.

##### Liver organoids

The liver’s remarkable regenerative capacity has made it a focus of organoid research [8, 9]. In a landmark study published in Nature, Takebe *et al.* demonstrated that induced pluripotent stem cell (iPSC)-derived liver buds containing hepatic endoderm, endothelial, and mesenchymal cells could self-organize into vascularized liver organoids that, upon transplantation into mice, connected to host vasculature within 48 h and exhibited liver-specific functions including albumin secretion and human-specific drug metabolism [9]. Bioprinting enables the fabrication of 3D mini-liver structures that secrete albumin and express hepatic markers [57]. The SWIFT (sacrificial writing into functional tissue) method has enabled the fabrication of organ-specific tissues with high cellular density (>10^8^ cells/mL) and embedded vascular channels capable of supporting perfusion [58].

##### Kidney organoids

iPSC-derived kidney organoids can recapitulate nephron structures including glomeruli, proximal tubules, and distal tubules [10, 59]. Homan *et al.* demonstrated that perfusion of kidney organoids under controlled flow significantly enhanced vascularization and tubular maturation, with increased expression of adult-specific transporters and improved nephrotoxicity responses to cisplatin [60]. Bioprinting enables the fabrication of kidney organoid arrays with controlled dimensions and perfusable tubular structures [34].

##### Cardiac organoids

Bioprinted cardiac constructs incorporating iPSC-derived cardiomyocytes can exhibit synchronized beating and respond to pharmacological agents [61]. Lee *et al.* developed the FRESH (freeform reversible embedding of suspended hydrogels) technique to bioprint collagen at unprecedented resolution, successfully fabricating patient-specific cardiac components including heart valves and a neonatal-scale human heart model with perfusable vasculature [62]. Noor *et al.* further demonstrated printing of personalized cardiac patches and hearts using patient-derived cells and ECM, achieving vascularized constructs that exhibited contractile function [63]. Preclinical studies with bioprinted cardiac patches using decellularized ECM bio-inks have shown improved cardiac function in rodent myocardial infarction models [64].

##### Neural organoids

Brain organoids have transformed neuroscience research [7]. For spinal cord injury, bioprinted constructs loaded with neural stem cells are being actively investigated using the ITOP technology [18]. The complexity of the central nervous system presents significant challenges, but progress continues toward clinical translation.

### Applications in plastic and reconstructive surgery

#### Skin and wound healing

Skin is the largest organ and the first line of defense against external insults. Large skin defects from burns, trauma, chronic wounds, or tumor resection present significant clinical challenges [65, 66]. 3D bioprinting has emerged as a powerful technology for creating skin substitutes that can accelerate wound healing and reduce scarring.

Bioprinted skin constructs typically incorporate multiple cell types to recapitulate the layered architecture of native skin [67]. The epidermal layer consists of keratinocytes that form a stratified epithelium providing barrier function, while the dermal compartment comprises fibroblasts within collagen-rich hydrogels that provide structural support and secrete ECM components. Endothelial cells can be incorporated to promote rapid vascularization after implantation, which is critical for the survival of thicker constructs [68]. More advanced constructs also include skin appendages such as hair follicles, sweat glands, and melanocytes for more complete functional skin regeneration.

*In situ* bioprinting represents a particularly compelling approach for wound healing, where cells and bio-inks are deposited directly onto the wound bed using handheld or robotic bioprinters [69, 70]. In a preclinical study using a porcine wound model, Albanna *et al.* demonstrated that *in situ* bioprinting of autologous dermal fibroblasts and epidermal keratinocytes directly onto full-thickness excisional wounds significantly accelerated wound closure compared to controls, with reduced contraction and accelerated re-epithelialization [71]. This approach eliminates the need for *in vitro* culture and enables patient-specific treatment [69]. Pourchet *et al.* developed a scaffold-free bioprinting approach using a dermal-epidermal construct that demonstrated proper stratification and differentiation after 26 days of culture, with histological features resembling native human skin [72]. For patients with pigmentation disorders, Min *et al.* successfully bioprinted melanocyte-containing skin constructs that achieved uniform pigmentation distribution [73].

Ouyang and colleagues developed a rapid printing approach for bio-inspired 3D tissue constructs that can recapitulate the complex anatomical structures and biological functions of native skin [74]. This technology addresses key challenges in existing bioprinting methods and demonstrates potential for fabricating implantable tissue constructs. Their approach incorporates multiple cell types in a spatially organized manner to achieve functional skin regeneration.

Recent advances include the incorporation of bioactive glass and other antimicrobial agents to prevent infection [68], as well as the development of prestress bioprinting techniques that can create skin with improved mechanical properties and enhanced angiogenesis [55].

#### Craniofacial reconstruction

Craniofacial defects resulting from trauma, congenital anomalies, or tumor resection require complex reconstructive procedures that can significantly impact patient quality of life [47, 75]. 3D bioprinting offers the ability to create patient-specific constructs that precisely match the complex anatomical geometry of craniofacial structures.

Medical imaging data (CT, MRI) can be directly translated into bioprinting instructions, enabling the fabrication of constructs that accurately fill defects with appropriate contours [47]. Applications in craniofacial reconstruction span a broad range of clinical scenarios. Calvarial bone defects resulting from trauma or craniotomy can be addressed with bioprinted bone constructs designed to match the complex cranial curvature [18]. Mandibular reconstruction following tumor resection benefits from patient-specific bioprinted mandible segments that restore both form and function [75]. Auricular reconstruction for patients with microtia or acquired ear defects represents a particularly compelling application given the complex 3D geometry of the external ear. Nasal reconstruction using bioprinted cartilage and soft tissue constructs addresses another area where precise anatomical matching is essential for acceptable aesthetic and functional outcomes.

The integration of bioprinting with patient-specific surgical planning represents a significant advance in reconstructive surgery, enabling truly personalized treatment approaches [47]. Lee *et al.* developed preclinically tested 3D bioprinted craniofacial constructs using materials with prior FDA clearance in other contexts, combined with osteogenic cells, demonstrating successful bone regeneration in critical-sized calvarial defects in preclinical models [47]. For auricular reconstruction, advances in cartilage bioprinting have demonstrated the feasibility of fabricating ear-shaped constructs that maintain anatomical shape and develop cartilage-specific ECM *in vivo*. These advances may support future personalized craniofacial reconstruction after validation of long-term safety, integration, and manufacturing consistency.

#### Adipose tissue engineering

Soft tissue reconstruction for breast reconstruction, facial rejuvenation, and body contouring represents a major application area in plastic surgery [70]. Current approaches using fat grafting suffer from unpredictable volume retention due to adipocyte necrosis. Bioprinted adipose tissue constructs offer the potential for improved outcomes through controlled architecture and vascularization.

Key considerations for adipose tissue bioprinting include appropriate cell source selection, scaffold design, and vascularization strategies. Adipose-derived stem cells serve as the primary cell source due to their capacity to differentiate into mature adipocytes while also contributing to vascularization through paracrine signaling. Scaffold design must balance high porosity to support adipogenesis with sufficient structural integrity to maintain volume over time. Vascularization is particularly critical for large-volume adipose constructs, as inadequate blood supply leads to central necrosis and unpredictable volume retention—the same limitation that affects conventional fat grafting procedures.

### Drug discovery and toxicology screening

#### Hepatotoxicity assessment

Drug-induced liver injury (DILI) is a leading cause of drug attrition and postmarket withdrawals [57]. Traditional hepatocyte cultures poorly predict DILI due to rapid loss of hepatic phenotype. Bioprinted liver organoid platforms maintain hepatic functions over extended periods and provide more predictive toxicity assessment.

Pharmaceutical companies have increasingly adopted 3D liver models for safety screening, with bioprinted platforms offering advantages in throughput and reproducibility [19].

#### Nephrotoxicity evaluation

The kidney is a major target of drug toxicity due to its role in drug excretion and high exposure to circulating compounds [59]. Bioprinted kidney organoids incorporating proximal tubule cells can model renal drug transport and nephrotoxic responses.

#### Cardiotoxicity screening

Cardiac safety is a critical component of drug development, with QT prolongation and arrhythmia risk requiring careful evaluation. Multisensor-integrated organs-on-chips platforms enable automated and continual *in situ* monitoring of organoid behaviors, including cardiac and hepatic function, providing comprehensive functional assessment for drug screening applications [76].

### Surgical planning and education

While distinct from therapeutic organoid applications, 3D bioprinted anatomical models incorporating patient-specific imaging data serve important clinical functions in surgical planning, training, and patient education [30]. These models can include color-coded tissue types and varying material properties to simulate surgical manipulation.

## Technological advances enabling clinical translation

### Vascularization strategies

**Figure 4:**
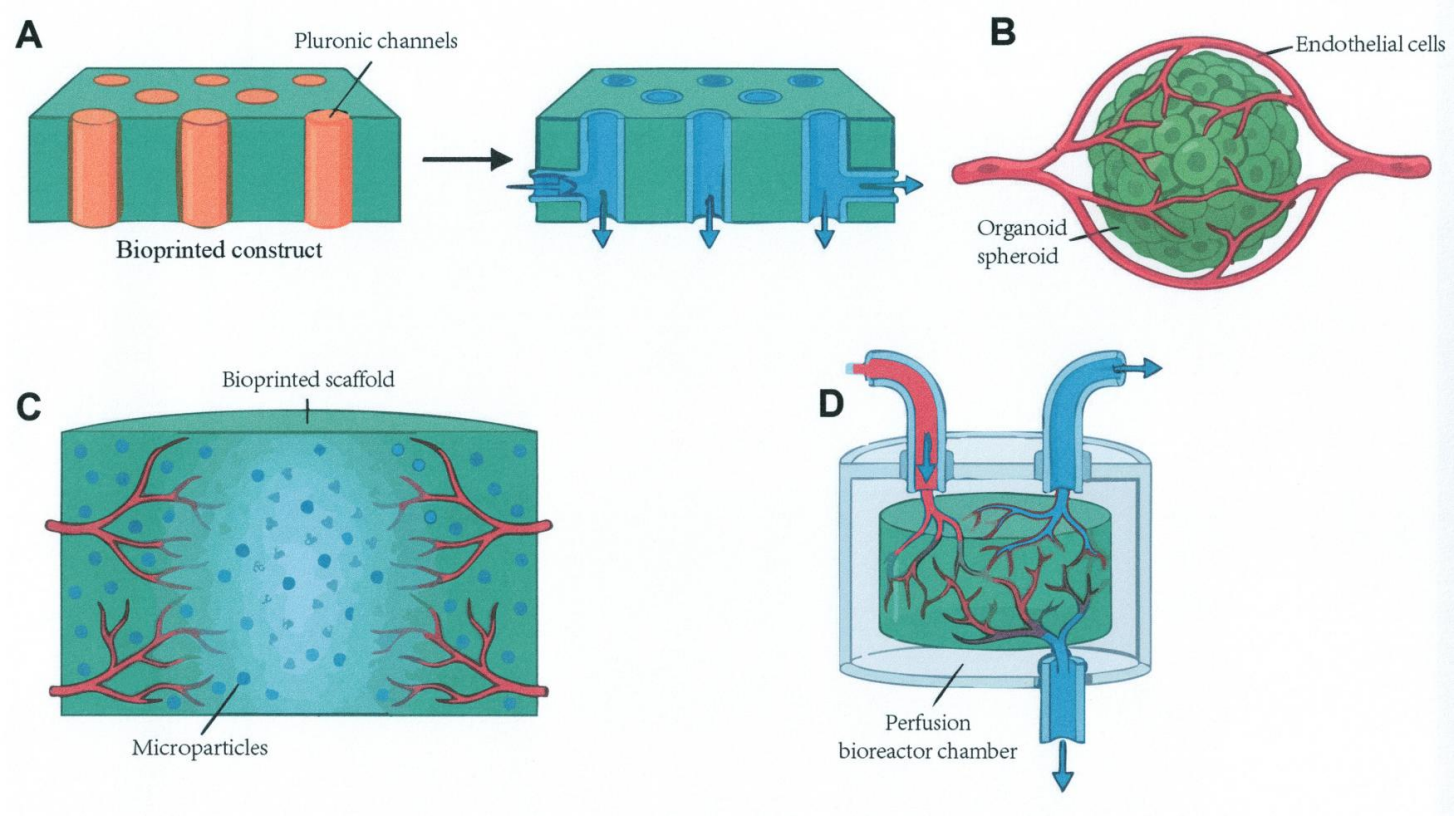
Vascularization strategies and technological advances. (**A**) Sacrificial templating approach for creating perfusable channels. (**B**) Coculture with endothelial cells for vascular self-organization. (**C**) Angiogenic factor incorporation and prevascularization strategies. (**D**) Prevascularization using a perfusion bioreactor chamber to generate *in vitro* vascular networks prior to organoid integration.

The lack of functional vasculature limits organoid size and viability, as diffusion alone cannot sustain metabolically active tissues beyond approximately 200 μm thickness (Figure 4) [15, 77]. Multiple complementary strategies have been developed to address this challenge. Sacrificial templating uses bioprinted sacrificial materials such as Pluronic F-127 or gelatin that are subsequently removed to create perfusable channels [14, 77]. Coculture approaches incorporate endothelial cells within bioprinted constructs, enabling self-organization into vascular networks [9, 58]. Additionally, sustained release of VEGF, FGF-2, and other pro-angiogenic factors can promote vascular ingrowth after implantation [33]. Finally, prevascularization strategies generate perfusable vascular networks *in vitro* before organoid integration, providing immediate perfusion capability upon assembly [60].

Despite these advances, significant limitations persist. Current vascularization strategies can generate perfusable macroscale channels, but achieving hierarchical vascular networks spanning arterioles to capillaries (<10 μm diameter) within bioprinted constructs remains elusive. Technologies such as the SWIFT method [58] and FRESH bioprinting [62] have demonstrated improved vascular channel resolution, yet anastomosis—the functional connection of engineered vessels with host vasculature upon implantation—remains a critical unsolved bottleneck that limits the clinical viability of thick bioprinted organoid constructs.

### Automation and scalability

Clinical translation requires scalable manufacturing processes that can produce organoids with consistent quality [30, 78]. Advances in robotic bioprinting systems, closed-loop process control, and integrated quality monitoring are enabling good manufacturing practice (GMP)-compatible organoid production. Automated multi-nozzle bioprinting platforms can now fabricate hundreds of organoid constructs per run with minimal operator intervention, reducing interbatch variability and labor costs [27]. Standardized bioprinter calibration protocols and real-time sensor feedback loops—monitoring parameters such as extrusion pressure, temperature, and cell-laden droplet volume—further enhance batch-to-batch consistency. However, scaling from laboratory prototypes to clinical-grade manufacturing remains a formidable challenge, particularly regarding cell expansion bottlenecks; achieving clinically relevant cell numbers (10^9^–10^10^ cells) requires bioreactor-based expansion systems that must maintain cell phenotype and genomic stability throughout prolonged culture [78]. Addressing these manufacturing challenges will be essential for reducing per-unit costs and enabling reimbursement-viable production of bioprinted organoid therapies.

### Quality control and standardization

Ensuring reproducibility and safety of bioprinted organoids requires robust quality control measures at multiple stages of the fabrication process [30, 78]. Cell source characterization and identity testing must confirm the phenotype, purity, and potency of input cell populations. Bio-ink sterility and endotoxin testing are essential to prevent contamination-related failures. Structural fidelity assessment through nondestructive imaging techniques such as micro-CT and optical coherence tomography enables verification that printed constructs match their design specifications. Functional validation assays specific to the target organoid type—such as albumin secretion for liver organoids or beating frequency for cardiac organoids—confirm biological performance. For stem cell-derived organoids, genomic stability monitoring through karyotyping or whole-genome sequencing is critical to detect potentially tumorigenic chromosomal aberrations that may arise during extended culture or the printing process.

### Critical evaluation: bioprinted organoids versus conventional approaches

A rigorous assessment of bioprinted organoids relative to conventional organoid systems reveals both significant advantages and persistent limitations across key translational metrics. In terms of structural fidelity, conventional organoids self-organize into structures that recapitulate native tissue architecture at the cellular level but with limited control over macroscale geometry and spatial organization of different cell types. Bioprinted organoids enable precise spatial patterning and controlled geometry; however, the printing process itself—including shear stress during extrusion and crosslinking-induced cytotoxicity—may disrupt the delicate self-organization that drives organoid formation. Current bioprinted constructs often lack the full cellular diversity and microarchitectural complexity observed in mature organs [11].

Regarding reproducibility, conventional organoids suffer from significant heterogeneity in size, shape, and cellular composition, with coefficients of variation typically ranging from 30% to 50%. Bioprinting substantially improves dimensional reproducibility, achieving coefficients of variation below 15% in organoid dimensions; however, variability in cell viability and functional maturation remains a challenge [30]. In translational relevance, bioprinted organoids offer clear advantages in scalability and standardization required for clinical applications and high-throughput drug screening. Nevertheless, the additional biomaterial components and crosslinking agents in bioprinted systems may introduce confounding variables not present in conventional organoid cultures. Scalability and physiological relevance therefore need to be interpreted together: bioprinting can improve standardized production, but clinical value depends on whether the imposed architecture improves function and maturation rather than geometry alone.

It is important to critically assess the evidence hierarchy supporting clinical translation. Patient-derived tumor organoids for drug sensitivity testing represent the most clinically validated application, with prospective studies supporting predictive performance in selected colorectal cancer settings, especially irinotecan-based metastatic colorectal cancer therapy and locally advanced rectal cancer chemoradiation [17, 38]. *In vivo* transplantation studies—including ITOP bone constructs, cardiac patches in rodent myocardial infarction models, and skin bioprinting in porcine wound models—constitute advanced preclinical evidence demonstrating therapeutic potential but have not yet entered human clinical trials [18, 71]. Many other reported applications, such as bioprinted liver mini-tissues and kidney organoid arrays, remain at the proof-of-concept stage, establishing technical feasibility but requiring extensive further development. Most bioprinted organoids also exhibit immature or fetal-like functional profiles, with cardiac, liver, kidney, and neural models often remaining substantially below adult or native tissue performance, highlighting the maturation gap that remains a central challenge [12, 61]. Thus, clinical translation is used here in a qualified sense: robust human-facing evidence is concentrated in patient-derived tumor organoid-guided treatment prediction, while implantable regenerative constructs remain largely preclinical.

## Challenges and future perspectives

### Current limitations

Despite remarkable progress, several challenges impede the widespread clinical adoption of 3D bioprinted organoids [27, 78]. A primary concern is functional maturation: bioprinted organoids often exhibit fetal-like phenotypes and require extended culture or additional stimuli—mechanical loading, electrical stimulation, and perfusion bioreactors—to approach adult tissue function; for example, many cardiac and liver organoid constructs still remain functionally immature relative to adult or native tissues [12, 61]. Closely related is the challenge of scale, as producing organoids of clinically relevant size remains limited by vascularization and nutrient diffusion constraints [15]. The complexity of native organs—which contain multiple cell types in precise spatial arrangements—is difficult to fully recapitulate through bioprinting [11]. Furthermore, most current bioprinted organoids lack resident immune cells, including tissue-resident macrophages, dendritic cells, and memory T cells, that are essential for physiologically relevant disease modeling and drug response prediction; incorporating immune components poses additional challenges because immune cells are particularly sensitive to the shear stresses and crosslinking conditions inherent in the printing process [37]. From a regulatory standpoint, the classification and approval process for bioprinted organoid products remains evolving, creating uncertainty for clinical translation [78]. Finally, current bioprinting processes and cell expansion requirements result in high costs that may limit accessibility [30].

These barriers should be interpreted together rather than independently: vascularization, immune incorporation, and maturation determine whether printed constructs can remain viable, physiologically informative, and durable beyond short-term *in vitro* assays.

### Integration with artificial intelligence

Artificial intelligence (AI) and machine learning are increasingly being integrated with bioprinting to optimize print parameters, predict organoid quality, and accelerate drug screening [79, 80]. Deep learning analysis of organoid morphology can identify subtle phenotypic differences indicative of drug responses or disease states.

Key applications of AI in bioprinting span the entire fabrication workflow [81, 82]. In bio-ink optimization, machine learning algorithms can predict optimal bio-ink compositions based on rheological properties, printability, and cell viability requirements, substantially reducing the need for time-consuming empirical optimization [83]. For process parameter optimization, AI-driven closed-loop control systems can automatically adjust printing parameters such as extrusion pressure, speed, and temperature in real time to maintain optimal print quality [81]. Quality assessment benefits from computer vision and image analysis algorithms that perform automated inspection of printed constructs, detecting structural defects and predicting functional outcomes [80]. In drug screening analysis, AI-powered high-content imaging analysis of organoid responses to drug libraries can accelerate drug discovery and improve prediction of patient responses [82].

The integration of AI and 3D bioprinting represents an emerging frontier for precision medicine, enabling more efficient development of patient-specific therapeutic solutions. At present, small training datasets, limited external validation, and uncertain long-term safety constrain clinical inference.

### Multiorgan systems

**Figure 5:**
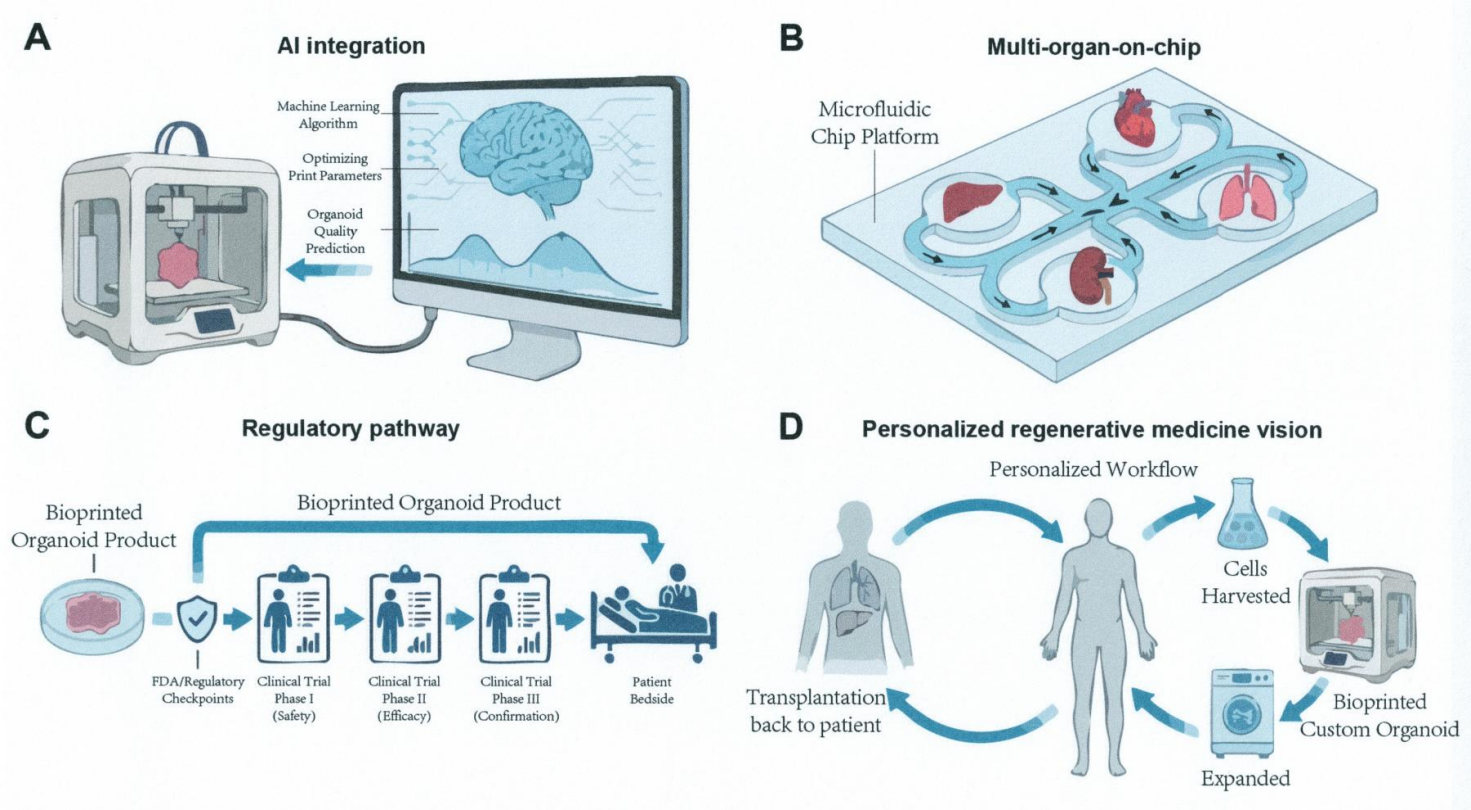
Future translational roadmap and key requirements. (**A**) 4D bioprinting with smart, stimuli-responsive biomaterials. (**B**) Multiorgan-on-a-chip systems for systemic drug evaluation. (**C**) Illustrative, product-specific regulatory considerations and GMP manufacturing requirements. (**D**) Vision for personalized regenerative medicine.

The development of bioprinted multiorganoid platforms connecting liver, kidney, heart, and other organoids through microfluidic circulation enables systemic drug evaluation and disease modeling (Figure 5) [76, 84]. These “body-on-a-chip” systems offer more comprehensive prediction of *in vivo* drug responses.

### 4D bioprinting and smart materials

The emergence of 4D bioprinting represents the next frontier in biofabrication, introducing the dimension of time to create dynamic, shape-morphing tissue constructs [85, 86]. Unlike static 3D printed structures, four-dimensional (4D) bioprinted constructs can change their shape, properties, or functionality in response to external stimuli such as temperature, pH, light, magnetic fields, or enzymatic activity.

Stimuli-responsive biomaterials, also known as “smart biomaterials,” form the foundation of 4D bioprinting [87, 88]. Shape memory polymers can recover their original shape upon exposure to specific stimuli such as temperature, light, or pH changes, enabling the fabrication of self-deploying implants. Thermo-responsive hydrogels undergo sol–gel transitions at physiological temperatures, allowing constructs to change properties in response to the body environment. pH-responsive polymers swell or contract in response to pH changes, offering applications in targeted drug delivery within organoid systems. Magnetically responsive composites incorporating magnetic nanoparticles enable remote actuation and spatial manipulation of constructs postimplantation, facilitating minimally invasive therapeutic interventions.

4D bioprinting has significant potential for creating self-folding tissue constructs that mimic developmental morphogenesis, developing smart implants that adapt to the physiological environment, and fabricating drug delivery systems with controlled release kinetics [89, 90]. This technology could support future regenerative-medicine strategies by enabling tissues that dynamically remodel and integrate with host tissues postimplantation, although long-term safety and functional validation remain unresolved.

### Regulatory considerations

The regulatory framework for bioprinted organoid products is still evolving [78, 91, 92]. Products may be classified as drugs, biologics, devices, or combination products depending on their intended use and composition. Engagement with regulatory agencies early in development is essential for navigating approval pathways.

Key regulatory considerations span product classification, manufacturing standards, safety and efficacy requirements, and ethical frameworks. Regarding product classification, bioprinted organoids may fall under different regulatory categories—biologics, medical devices, or combination products—depending on their composition and intended use, with classification varying across jurisdictions (FDA, European Medicines Agency, National Medical Products Administration) [91, 92]. Specific applicable standards include ISO/ASTM 52900:2021 for additive manufacturing terminology, ISO 10993 series for biological evaluation of medical devices, ASTM F2739-19 for quantifying cell viability within biomaterial scaffolds, ASTM F3354-19 for evaluating ECM decellularization processes, and ISO 13485:2016 for quality management systems [93]. The FDA’s 2017 guidance on Technical Considerations for Additive Manufactured Medical Devices is most directly relevant to device components or device-led combination products that include additive manufacturing, whereas the EU Advanced Therapy Medicinal Products Regulation (EC) No. 1394/2007 provides a framework for relevant advanced therapy products; final applicability is product-specific. Safety and efficacy requirements demand rigorous preclinical studies with particular attention to tumorigenicity for stem cell-derived products and immunogenicity for allogeneic products [94]. Ethical considerations encompass informed consent for cell donation and organoid derivation, tissue ownership questions, the implications of creating increasingly complex human tissue models, and equitable access to these advanced therapies [91].

Despite these challenges, the regulatory landscape is gradually maturing, with agencies developing specific guidance for tissue-engineered products and providing pathways for innovative therapies [92]. Manufacturing scale-up from bench-top prototyping to GMP-compliant production presents formidable challenges, including cell expansion bottlenecks to achieve clinically relevant cell numbers (10^9^–10^10^), bio-ink sterilization without compromising bioactivity, cold-chain logistics for living products, batch release testing requirements, and cost-effectiveness analyses essential for reimbursement approval. The International Council for Harmonisation Q5A(R2) and Q5D guidelines may inform viral-safety and cell-substrate controls for products using relevant cell substrates or biological starting materials, but direct applicability is product-specific. Thus, the practical translational threshold is not only proof of fabrication but also validated classification, GMP release criteria, cell-source traceability, cost control, and equitable access. An overview of emerging technologies and regulatory considerations is provided in Table 3.

**Table 3:** Emerging technologies in organoid bioprinting: 4D bioprinting, AI integration, and regulatory considerations.

Technology	Key features	Potential impact	Current challenges
4D bioprinting			
Shape memory polymers	Recover original shape upon stimuli	Self-folding tissue constructs	Limited biocompatibility
Thermo-responsive hydrogels	Sol–gel transition at body temperature	Smart drug delivery	Precise control of transition
Magnetic composites	Remote actuation capability	Minimally invasive deployment	Long-term safety
AI integration			
ML for bio-ink optimization	Predict printability and cell behavior	Reduced trial and error	Training data availability
Real-time quality control	Monitor printing parameters	Improved reproducibility	Sensor integration
Generative design	Optimize scaffold architecture	Enhanced functionality	Computational resources
Regulatory framework			
Product classification	Biologics, devices, or combination	Clear approval pathway	Case-by-case determination
GMP manufacturing	Validated processes, QC testing	Clinical-grade products	High implementation cost
Ethical considerations	Tissue ownership, informed consent	Public trust	Evolving guidelines

## Conclusion

The convergence of 3D bioprinting technology and organoid biology represents a major advance for biomedical modeling and translational research [1, 12]. Bioprinted organoids expand capabilities for personalized cancer-model testing, regenerative-medicine research, drug discovery, and related applications. The field has witnessed remarkable progress, from the pioneering demonstrations of tissue printing to the current era of patient-derived organoid biobanks and AI-optimized biofabrication [95].

In the realm of precision medicine, patient-derived tumor organoids have shown clinical utility most clearly in treatment-response prediction, with prospective studies supporting their role in therapeutic decision-making [96, 97]. In regenerative medicine, preclinical advances in vascularization strategies, bone and cartilage organoid engineering, and skin bioprinting are addressing barriers that have historically limited translation [43, 49].

Emerging technologies including 4D bioprinting with smart materials and AI-powered optimization are opening new frontiers in the field [80, 85]. These approaches may enable more sophisticated tissue constructs that respond to physiological cues and improve fabrication workflows, provided that safety and external validation improve.

While significant challenges remain in achieving vascularization, maturation, scale, and regulatory approval, the rapid pace of technological innovation continues to push the boundaries of what is possible [2, 27]. The clinical translation of 3D bioprinted organoids will require continued interdisciplinary collaboration among engineers, biologists, clinicians, and regulatory scientists [78, 92]. If these technologies mature through prospective validation and standardized manufacturing, selected applications could contribute to more predictive disease models, more effective therapy selection, and carefully evaluated regenerative treatments.

Future clinical impact will depend on prospective validation, standardized manufacturing, immune and vascular integration, long-term safety, cost control, and reimbursement feasibility. With these constraints addressed, selected applications may move from technical feasibility toward responsible clinical evaluation [20, 98].
